# Supplementary material for: Reconciling Mining with the Conservation of Cave Biodiversity: A Quantitative Baseline to Help Establish Conservation Priorities
Source: PLoS One. 2016 Dec 20;11(12):e0168348. doi: 10.1371/journal.pone.0168348 (PMC5173368; doi:10.1371/journal.pone.0168348)
Supplement: S1 Dataset — (ZIP) [file pone.0168348.s002.zip › Taxa/Serra Norte/SN_2013/N4E/Lista N4E-32.pdf]

CAVIDADE N4E-0032							
**Classe**	**Ordem**	**Fam/Outros**	**Gên/Outros**	**Espécie**	**S-U**	**U-S**	Zon.
Arachnida	Acari			jovem	X		E
Arachnida	Araneae	Araneidae	*Alpaida*	sp.1		X	E
Arachnida	Araneae	Corinnidae		jovem		1	E
Arachnida	Araneae	Ctenidae		jovem		1	E
Arachnida	Araneae	Dipluridae		jovem		1	E
Arachnida	Araneae	Pholcidae	*Mesabolivar*	aff. *cambridgei*	X	X	E
Arachnida	Araneae	Salticidae		sp.6	X	X	E
Arachnida	Araneae	Scytodidae	*Scytodes*	jovem	1		E
Arachnida	Araneae	Segestriidae	*Ariadna*	sp.		X	E
Arachnida	Araneae	Theridiidae	*Theridion*	sp.		X	E
Arachnida	Araneae	Theridiosomatidae	*Plato*	sp.		X	E
Arachnida	Opiliones	Sclerosomatidae	Gagrellinae	sp.		X	E
Arachnida	Pseudoscorpiones	Chernetidae	*Spelaeochernes*	sp.1	X		E
Chilopoda	Geophilomorpha			sp.		X	E
Chilopoda	Scolopendromorpha	Scolopocryptopidae	*Newportia*	sp.		X	E
Chilopoda	Scutigeromorpha	Pselliodidae	*Sphendononema*	*guildingii*		X	E
Entognatha	Collembola	Paronellidae		sp.1	X	X	E
Insecta	Blattodea			jovem		X	E
Insecta	Coleoptera	Dytiscidae		sp.		X	E
Insecta	Coleoptera	Tenebrionidae	Alleculinae	sp.		X	E
Insecta	Diptera	Cecidomyiidae	Cecidomyiinae	sp.	X		E
Insecta	Diptera	Dolichopodidae		sp.	X		E
Insecta	Diptera	Psychodidae	*Pintomyia*	*gruta*		X	E
Insecta	Diptera	Tethinidae		sp.		X	E
Insecta	Heteroptera	Cydnidae		sp.		X	E
Insecta	Hymenoptera	Formicidae	*Camponotus*	sp.		X	E
Insecta	Hymenoptera	Formicidae	*Dolichoderus*	aff. *bispinosus*	X	X	E
Insecta	Hymenoptera	Formicidae	*Ectatomma*	*edentatum*			E
Insecta	Hymenoptera	Formicidae	*Pachycondyla*	sp.2	X		E
Insecta	Hymenoptera	Formicidae	*Pheidole*	gr. *flavens*		X	E
Insecta	Isoptera	Termitidae	*Nasutitermes*	sp.	X	X	E
Insecta	Lepidoptera	Tineoidea		jovem		X	E
Insecta	Orthoptera	Gryllidae	Eneopterinae	sp.	2		E
Insecta	Psocoptera			jovem	X		E
Malacostraca	Isopoda	Philosciidae		sp.2	X		E
Symphyla		Scutigerellidae	*Hanseniella*	sp.		X	E
